# Attitudes of Swedish primary care physicians toward GLP-1 receptor agonists for overweight and obesity: a cross-sectional survey

**DOI:** 10.1186/s12875-026-03488-y

**Published:** 2026-08-05

**Authors:** Isabelle Sundell, Björn Wettermark, Anders Sundström, Mats Martinell, Björn Ericsson, Ulf Lindahl, Hussein N. Rubaiy, Maria Palmetun Ekbäck

**Affiliations:** 1 Faculty of Pharmacy, Uppsala University, Uppsala, Sweden; 2 Uppsala Monitoring Center, Uppsala, Sweden; 3 Department of Public Health and Caring Sciences, Uppsala University, Uppsala, Sweden; 4 Gävleborg Drug & Therapeutics Committee, Gävle, Sweden; 5 Västernorrland Drug & Therapeutics Committee, Härnösand, Sweden; 6 Department of Laboratory Medicine, Division of Clinical Pharmacology, Karolinska Institute, Karolinska University Hospital, Stockholm, Sweden; 7 School of Medical Science, Örebro University, Örebro, Sweden; 8 Dermatology Department, Örebro University Hospital, Örebro, Sweden; 9 Department of Pharmacology and Therapeutics, Örebro County Council , Örebro University Hospital, Södra Grev Rosengatan, Örebro, 70185 Sweden

**Keywords:** Primary health care, Glucagon-like peptide-1 receptor agonists, Obesity, Diabetes, Sweden, Survey, Cross sectional study

## Abstract

**Background:**

Obesity is a growing global health burden, previously with few effective treatments. Glucagon-like peptide-1 receptor agonists (GLP-1 RAs) were originally approved for diabetes but are increasingly prescribed for treatment of overweight and obesity. The increasing expenditure has led to ethical debates surrounding the utilization of these drugs. Few studies have investigated the attitudes to GLP-1 RAs among prescribing physicians. This study aimed to assess the attitudes to GLP-1 RAs for treatment of overweight and obesity among Swedish primary care physicians.

**Methods:**

This was a cross-sectional survey with descriptive quantitative analysis and thematic analysis of free-text responses. A mix of multiple-choice questions and rating scales, including the Likert scale was employed. The study was conducted in October 2024 among physicians working in primary care in the Swedish regions of Gävleborg, Uppsala, Västernorrland, Västmanland and Örebro. The survey was distributed through the Drug and Therapeutics Committees (DTCs) in the regions.

**Results:**

The 190 responses yielded a response rate of 28%. Of these, 163 responses could be included in the final analysis. Almost all respondents (98%) agreed that GLP-1 RAs are effective drugs for treating obesity, and 91% believed they will be important for obesity treatment in the future. Main identified barriers for prescribing GLP-1 RAs were high patient co-payment (69%) and low drug availability due to supply shortages at the time of conducting this survey (86%).

**Conclusion:**

Physicians working in primary care in Sweden consider GLP-1 RAs effective for treating obesity and believe these drugs will play an important role in obesity treatment in the future. These findings should be interpreted in the light of the low response rate and possible self-selection bias.

**Supplementary Information:**

The online version contains supplementary material available at 10.1186/s12875-026-03488-y.

## Introduction

Obesity represents a rapidly escalating global health crisis, closely associated with an increased risk of numerous chronic conditions such as type 2 diabetes, cardiovascular disease, and certain cancers [1, 2]. In Sweden, the prevalence has tripled since 1980 and in 2022 more than half of the Swedish population aged 16–84 (52%) were either overweight or obese [2, 3]. A report from the Swedish capital region of Stockholm showed that obesity was the eighth most commonly recorded chronic diagnosis among individuals who visited the primary care in 2018 [4]. However, studies suggest that there is an underreporting with less than 40% of all individuals with obesity being diagnosed [5].

Swedish guidelines for obesity treatment promote a multidisciplinary approach and the interventions include physical activity, dietary changes, behavioural therapy, pharmacological treatment and bariatric surgery [6]. Lifestyle interventions are the preferred treatment method for mild to moderate forms of obesity, but they have shown to be insufficient in promoting sustainable long-term weight loss results for most patients [7].

The development of pharmacological treatments of obesity has been full of failures and there are several historical examples of obesity medications being withdrawn from the market after regulatory approval due to safety concerns such as adverse cardiovascular effects, increased suicidal risks, risk for abuse or cancer [8, 9]. During the last decade, GLP-1 RAs have been approved for obesity treatment since they have been shown to be effective for promoting weight loss in clinical trials [10, 11]. While previously approved obesity medications were associated with numerous adverse physical and psychological effects, these new agents seem comparatively safe. Systematic reviews have found the most common adverse events to be nausea, vomiting, constipation and diarrhoea, all of which were mild to moderate in severity [11, 12].

In recent years, GLP-1 RAs have become increasingly discussed in the medical debate and on social media, and its use is rapidly increasing globally [13–17]. A recent study showed an exponential increase in the use of GLP-1 RAs for obesity treatment also in Sweden [18]. The rapid increase in demand for GLP-1 RAs, in combination with a limited production capacity, resulted in widespread drug shortages of GLP-1 RAs. As a result, the Swedish Medical Products Agency (SMPA) made an appeal to all prescribing physicians to restrict prescribing of Ozempic^®^ to patients with T2DM, to secure availability of these medications for those who need them the most [19]. These appeals did not pass without criticism, and researchers in the obesity field have stated that both obesity and T2DM are chronic diseases which can affect the patients in different ways, and that every patient should be managed individually [20].

Previous studies investigating knowledge, experiences and/or perceptions of GLP-1 RAs for treating T2DM among physicians have been conducted in the UK [21], Canada [22], Saudi Arabia [23], and Belgium [24]. Few studies, however, have explored the attitudes to GLP-1 RAs for treatment of overweight and obesity among prescribing physicians. One survey study conducted in the US in 2023 investigated healthcare professionals’ experience of obesity treatment and their understanding of different obesity medications [25]. The study found that less than 50% of the patients with obesity were prescribed obesity medications, and the greatest barriers to treatment were medication cost and lack of insurance coverage. Other perceived barriers were low patient engagement/adherence and inadequate time/staff [25]. These results, however, are not specific for the class of GLP-1 RAs, and therefore, there is a need for further studies specifically aimed at investigating the attitudes surrounding the use of GLP-1 RAs for treating overweight and obesity among prescribing physicians.

## Materials and methods

### Study design

This was a cross-sectional survey with descriptive quantitative analysis and thematic analysis of free-text responses. The study aimed to assess the attitudes surrounding the utilization of GLP-1 RAs for treatment of overweight and obesity among primary care physicians in Sweden and was conducted in October of 2024. A questionnaire was developed using the REDCap electronic data capture tools [26] and sent out to all physicians working in primary care in five regions of Sweden; Gävleborg (*n* = 80), Uppsala (*n* = 112), Västmanland (*n* = 108), Västernorrland (*n* = 77) and Örebro (*n* = 300). A total of 677 physicians working in primary care were invited. The questionnaire consisted of questions about characteristics of the respondent and his/her attitudes about obesity as an illness, and questions aimed at assessing the respondents´ attitudes toward treatment with GLP-1 RAs. A mix of multiple-choice questions, rating scales and dichotomous questions was employed. One of the questions provided the possibility to give a free text response, and a thematic analysis was conducted on those responses. Coding and analysis were performed by the first author and discussed with three other authors. All authors were then involved in discussing the findings from the thematic analysis. All questionnaire items provided the answer option of “I do not know/I do not want to answer/I do not have an opinion”. For some of the questionnaire items, respondents were asked to elaborate their responses if one or more specific answer option(s) were selected. For some of the demographic questions, respondents were asked to specify if they selected the option “other” to the question.

A Likert scale was employed for five of the questionnaire items. For each Likert questionnaire item, which was formulated as a statement, the respondents presented their viewpoint by selecting an answer on a symmetric scale ranging from “strongly disagree” to “strongly agree” (1 = strongly disagree; 2 = disagree; 3 = neither agree nor disagree; 4 = agree; 5 = strongly agree; 6 = I do not know) [27]. Two of the Likert scale items used reversed score, as to reduce bias of the study [28].

### Survey development

The questionnaire was developed in several steps. Prior to the questionnaire development, a thorough review of previously published research in the area was conducted to obtain information about what had already been done in this area of research [29]. Questionnaire items were suggested by the first author and then further developed and improved during several discussions with the other authors. During development of the questionnaire, some questions were removed or replaced, and several were reformulated. All questionnaire items were formulated as to avoid confusion or leading to bias [30–33]. A pilot test with further improvement was then conducted through a focus group consisting of three primary care physicians, one information pharmacist and the chairman of a Drug and Therapeutics committee. People in the focus group completed the questionnaire, reported how long it took to complete and evaluated it based on comprehensibility, face validity, content validity and construct validity. Following the pilot test, the questionnaire was further revised, including the addition of a free-text response option to question 8, to reduce the risk of response misinterpretation. However, no further validation of the questionnaire was conducted. The final questionnaire consisted of a total of 20 items, including 11 GLP-1 RA items. The questionnaire was considered to take around 5–10 min to complete (see appendix 1).

### Population

Study participants were recruited from the Swedish regions of Gävleborg, Uppsala, Västernorrland, Västmanland and Örebro. Participants were eligible if they worked in primary care in Sweden as a physician undergoing specialist training (ST), as a specialist physician in primary care or as a licensed physician in primary care. The managers of all primary care clinics in the regions were contacted by email between October 8th and October 18th. In Örebro, the chairman of the DTC forwarded the author’s invitation email directly to physicians working in primary care in the region on October 10th. The email list used consisted of around 300 people. However, another source implied that there were only 110 primary care physicians in Örebro at the time, indicating that the email invitation also reached physicians who were temporarily stationed in Örebro. The managers received an invitation email with information about the study, an invitation letter and a link, as well as a QR-code to the questionnaire that they were asked to forward to their physician colleagues at their clinic (see appendix 2). The managers were also asked to share with the authors how many physicians received the survey, to keep track of the total number of physicians who received it. Thereafter, two reminders were sent out with approximately one-week intervals, except in Gävleborg, where only one reminder was sent out.

### Data collection and analysis

Data were collected between October 8th and November 3rd of 2024 and analysed using descriptive statistics and confidence intervals based on differences in proportions. For all questionnaire items, the response distribution was calculated as frequencies and proportions, and the results were illustrated in tables and figures. For analysing potential differences between gender and private/regional primary care, a 95% confidence interval was calculated based on the differences in proportions between the two groups, i.e. men and women, and private and regional primary care, respectively. For Likert scale items, when analysing differences between groups, the top two boxes (‘agree’ and ‘strongly agree’) and bottom two boxes (‘disagree’ and ‘strongly disagree’) were summed and combined into “agree” and “disagree”, respectively. Data were extracted from the REDCap survey tool and analysed using Microsoft Excel version 16.90.2. Qualitative data obtained from free text responses to item 8 was analysed using an inductive thematic analysis, with a focus on semantic interpretation [34].

### Ethical considerations

Primary care physicians or physicians working in primary care were invited to participate in this study and were informed about the aim of the study, length of the survey and how their answers would be utilized. They were also informed that their responses were collected anonymously, that the participation was voluntary, and that they should only answer questions they were comfortable answering. Since the survey was anonymous and obtained no sensitive personal data, an application to the Ethical review Authority (ERA) was not required, in accordance with Swedish legislation (law nr: 2003:460).

## Results

### Study participants

**Fig. 1:**
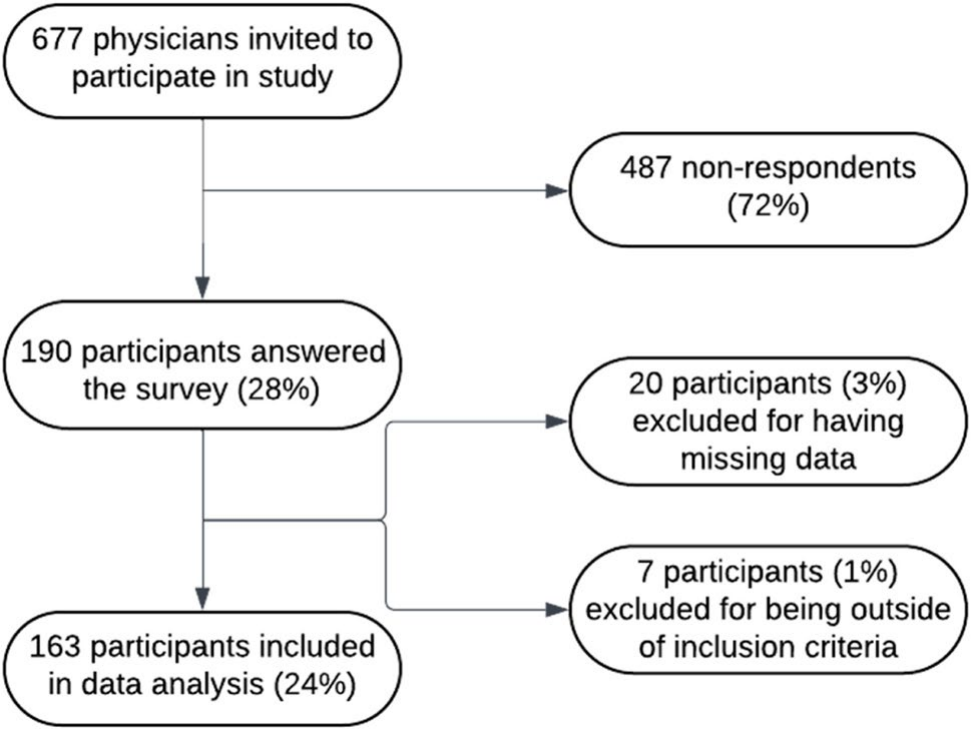
Number of participants in study, including number invited, response rate and reasons for exclusion

The survey received a total of 190 responses which yielded a response rate of 28%, which is a low response rate. The response rates varied between regions from 19% in Örebro to 39% in Västmanland. Of the 190 responses, 163 participants were included in the final analysis (Fig. 1). A total of 20 responses were incomplete and had missing answers for items 10–20. Seven participants were excluded from the study due to not meeting the inclusion criteria, as shown in Fig. 1, and these consisted of 3 junior training physicians, 2 assistant physicians, 1 quality coordinator and 1 member of a DTC. The subgroup findings should be interpreted as exploratory.

Of the 163 respondents included in the study, 61% (*n* = 100) were women and 37% (*n* = 61) were men (2 respondents answered ‘other’). Most respondents (62%) were specialists in general practice and had worked for more than 10 years as a physician (63%). Most of the respondents worked in regional primary care (60%) and the region contributing with the largest number of respondents was Örebro (32%), as seen in Table 1.

**Table 1:** Characteristics of Swedish physicians working in primary care responding to a survey on obesity and GLP-RAs

Variable	Participant Characteristics	Number of participants (%)
Gender	Men	61 (37%)
	Women	100 (61%)
	Other	2 (1%)
Profession	Resident in general practice	57 (35%)
	Specialist in general practice	101 (62%)
	Specialist in other area	2 (1%)
	Licensed physician in primary care	3 (2%)
Time worked as a physician^1^	0–5 years	28 (17%)
	6-10 years	32 (20%)
	More than 10 years	102 (63%)
Region	Gävleborg	20 (12%)
	Uppsala	37 (23%)
	Västmanland	36 (22%)
	Västernorrland	18 (11%)
	Örebro	52 (32%)
Practice ownership	Public primary care clinic	98 (60%)
	Private primary care clinic^2^	65 (40%)
Frequency of seeing patients in need of obesity treatment	Never	1 (1%)
	Once per month or more seldom	22 (14%)
	Several times per month	78 (48%)
	Once per week	14 (9%)
	Several times per week	36 (22%)
	Every day	11 (7%)
	I do not know	1 (1%)

^1^Missing = 1

^2^Private clinics which have contracts with the regions (as the ones included in this study have) operate under the same conditions as regional clinics

### Attitudes surrounding obesity

Most respondents (*n* = 132, 83%) answered that obesity is a chronic disease. There was a statistically significant difference between men (72,1%) and women (86,0%), where female physicians considered obesity to be a chronic disease to a higher degree (C.I 0,7–27,0) (see appendix 4, Table A1). There were some differences in answers between primary care physicians working in regional primary care or private primary care, but it did not reach statistical significance (see appendix 4, Table A2). Most respondents (*n* = 95, 59%) considered obtaining a healthier weight to be mainly the patient’s responsibility, although 28% (*n* = 45) believed that the responsibility is equally shared between the patient and the primary care physician, and 9% (*n* = 14) believed the patient is fully responsible.

**Fig. 2:**
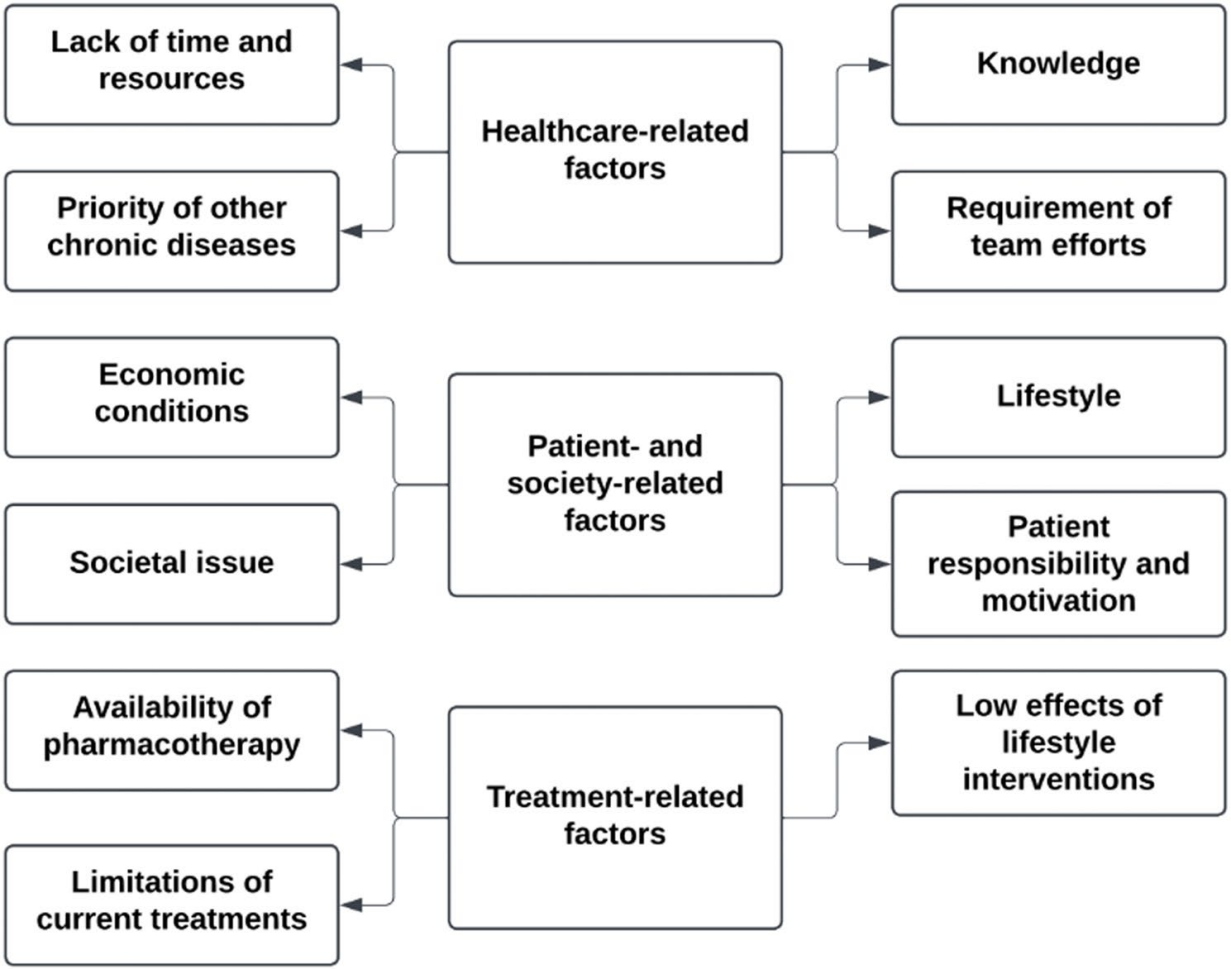
Themes and codes established from the thematic analysis

A majority of respondents either agreed (*n* = 74, 45%) or strongly agreed (*n* = 37, 23%) that primary care physicians have the prerequisite to help patients with obesity achieve a healthier weight. 19% disagreed with the statement (*n* = 31), 9% strongly disagreed (*n* = 14) and 4% neither agreed nor disagreed (*n* = 7). A free text motivation to this question could be provided, and a total of 90 free text responses were obtained. From the thematic analysis conducted on those responses, three different themes were established describing aspects of primary care physician’s view on obesity management: (1) Healthcare-related factors, (2) Patient and society-related factors, and (3) Treatment-related factors, which are shown in Fig. 2.

Lack of time and/or resources, prioritization of other chronic diseases and requirements of team efforts from multiple professions was stated by many respondents as limiting factors for optimal obesity treatment within primary care.

“There is not enough time to help 50% of the Swedish population lose weight, it would sideline many other patient categories. It is not certain that all patients with obesity have an unhealthy weight.”

Limitations and low effectiveness of currently available treatments, such as lifestyle interventions and older anti-obesity medications, in combination with low availability and high cost of effective pharmaceutical treatments like GLP-1 RAs, were stated by respondents as other limiting factors.

“Mysimba and Orlistat are not used very much, and GLP-1 RAs and its associated shortage situation means that we do not prescribe it based solely on weight indication and extremely sparingly for overweight diabetics.”

The economic condition of the patient was also brought up as important for determining treatment alternatives and outcome, due to the cost of pharmaceuticals not included in the benefit.

“We can prescribe GLP-1 RAs, but since they are not included in the benefit, they are not available for everyone.”

Respondents also stated that obesity is a societal issue that primarily needs to be dealt with on a population level, and that patient motivation and lifestyle factors are hard to target within primary care alone.

### Attitudes surrounding GLP-1 RAs

**Fig. 3:**
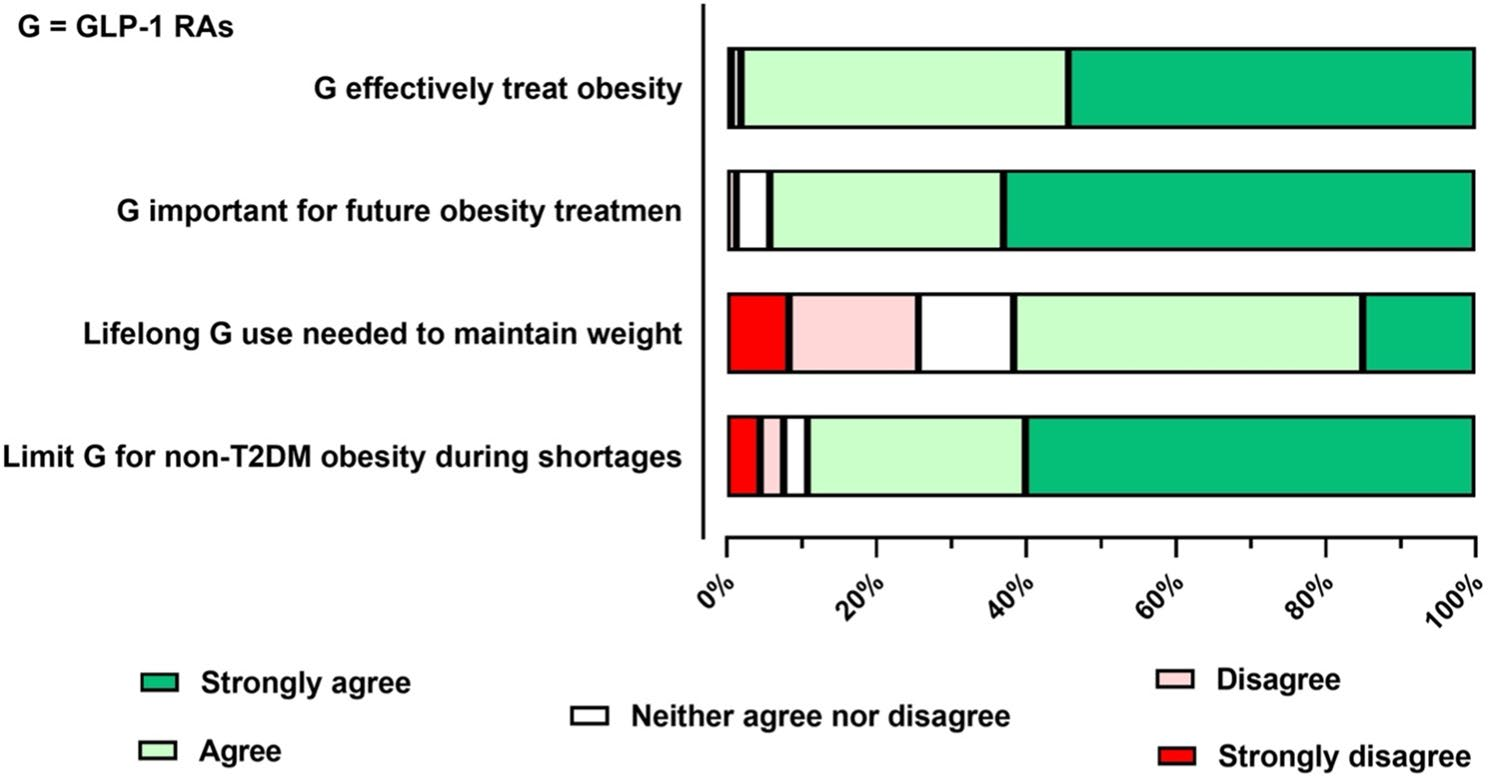
Response distribution among primary care physicians for the Likert scale questions on GLP-1 RAs

A large majority of respondents either strongly agreed (54%) or agreed (44%) that GLP-1 RAs are effective drugs for weight loss among patients with obesity who have not achieved sufficient weight loss from lifestyle interventions alone (Fig. 3). Likewise, most respondents (91%) either strongly agreed (61%) or agreed (30%) that GLP-1 RAs will likely play an important role in the treatment of obesity in the future. To the statement that GLP-1 RAs require a lifelong treatment to maintain a healthier weight, the answer option that yielded the highest proportion of responses was ‘agree’ (38%), followed by ‘I do not know/I do not have an opinion’ (18%) and ‘disagree’ (14%). Most respondents strongly agreed (58%) and 28% agreed to the statement that prescription of GLP-1 RAs to patients with obesity without T2DM should be limited when availability of these drugs is low.

**Fig. 4:**
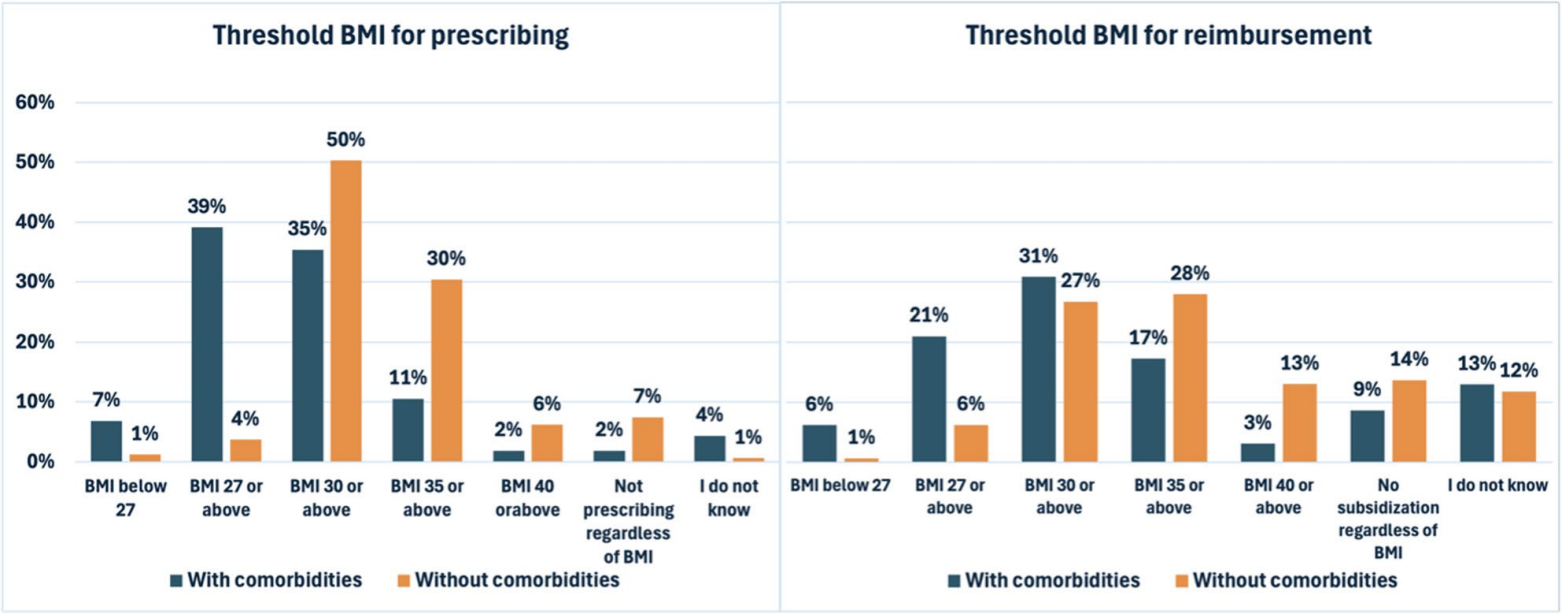
Response distribution among primary care physicians for the following questions: “At what minimum BMI would you consider prescribing GLP-1 RAs to patients WITH/WITHOUT weight related comorbidities?”, and: “At what minimum BMI would you consider that GLP-1 RAs should be subsidized for patients WITH/WITHOUT weight related comorbidities?”

Half of respondents would consider prescribing GLP-1 RAs to patients without weight related comorbidities at a minimum BMI of 30 or above, and 30% of respondents answered a minimum BMI of 35 or above (Fig. 4). For patients with weight related comorbidities, the most frequent answers were that they would consider prescribing GLP-1 RAs at a BMI of 27 or above, followed by a BMI of 30 or above, as shown in Fig. 4. There was a significant positive association where female physicians to a higher degree considered prescribing GLP-1 RAs to patients with weight related comorbidities at a lower BMI of 27 or above (C.I 2,2–32,0) (see appendix 4, Table A1).

Most respondents answered either a minimum BMI of 35 or above or a minimum BMI of 30 or above when asked at what minimum BMI they would consider that GLP-1 RAs should be subsidized for patients without weight related comorbidities (Fig. 4). For patients with weight related comorbidities, the most frequent answer was a BMI of 30 or above.

**Fig. 5:**
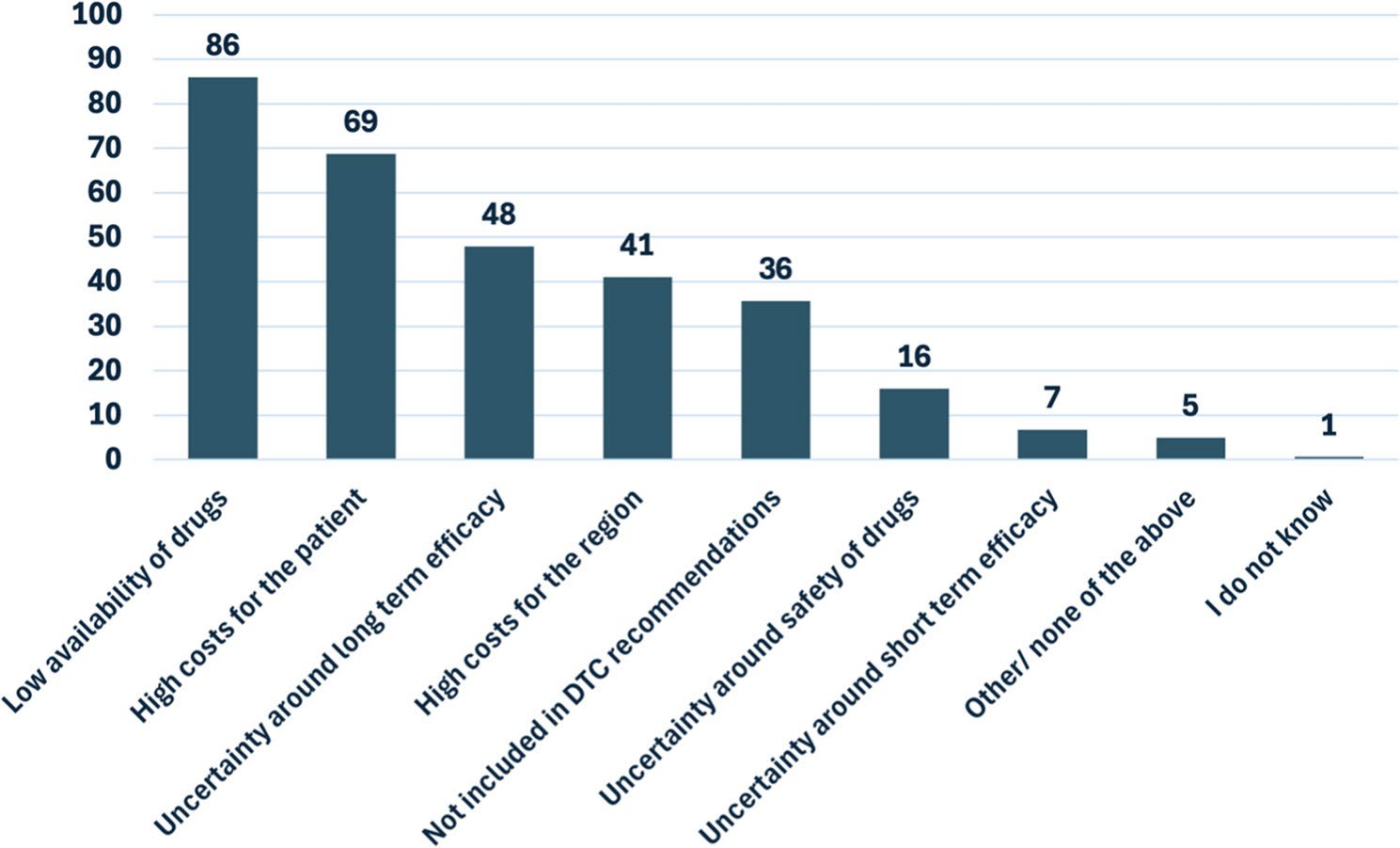
The proportion of respondents (%) that stated the following barriers for them to consider prescribing GLP-1 RAs for treatment of obesity, in order of highest to lowest: (1) Low availability of drugs, (2) High costs for the patient, (3) Uncertainty around long term efficacy of drugs for weight reduction, (4) High costs for the region, (5) Drugs are not included in the recommendations from the DTC, (6) Uncertainty around safety of drugs, (7) Uncertainty around short term efficacy of drugs for weight reduction, (8) Other/ none of the above, (9) I do not know

A majority of respondents stated low availability of drugs and high costs for the patient as barriers for them to consider prescribing GLP-1 RAs for obesity. Almost half of respondents also stated uncertainty around long term efficacy of drugs for weight reduction as a barrier (Fig. 5).

## Discussion

To the best of our knowledge, this is the first Swedish study investigating self-reported attitudes surrounding the utilization of GLP-1 RAs for treatment of overweight and obesity among primary care physicians in Sweden. The most important findings include that physicians working in primary care almost unanimously believe GLP-1 RAs to be effective drugs for obesity treatment. The types of drugs they would consider prescribing and at what minimum BMI it would be considered align with current guidelines. At the time of conducting this study, there were several barriers limiting the prescribing of GLP-1 RAs for weight loss, of which the most common were low drug availability and high patient costs. Supply constraints may have changed since the survey period, but high patient cost is still a barrier. Despite this, physicians working in primary care in the middle part of Sweden believe that GLP-1 RAs will play an important role in the treatment of obesity in the future.

The comparison to previous studies on obesity medications is indirect as earlier studies also assess older anti-obesity drugs. However, our findings indicate more positive attitudes toward these drugs, suggesting that GLP-1 RAs are perceived as safer and more effective compared to older generations of weight loss drugs. In the ACTION study by Kaplan et al., only 30% of healthcare providers believed that obesity medications were completely effective for weight management (2018). Although the recognition of obesity as a chronic disease has increased in recent years, stigma still constitutes a barrier for optimal obesity treatment [2]. Another Swedish survey found a positive association between physicians’ knowledge about obesity and adherence to obesity guidelines [2]. The study also found that more than half of primary care physicians (58%) did not consider obesity pharmacotherapy to be effective for weight loss among patients with moderate obesity (BMI of 30–39.9 kg/m2), further demonstrating the shift in attitudes in recent years [2].

This study’s findings suggest that physicians working in primary care are somewhat uncertain about the long-term effects of GLP-1 RAs, and half viewed lifelong treatment as necessary for sustained weight management. These results are consistent with Tchang et al., who state that pharmacological treatment of obesity should be considered a lifelong treatment, since obesity is a chronic disease and discontinuation of the treatment often leads to weight regain [35]. This also highlights the importance of educating patients on implementing a healthier lifestyle to decrease the risk of weight regain. Only 16% of respondents stated uncertainty around the safety of the drugs as a barrier for prescribing, implying that GLP-1 RAs, unlike previous obesity medications, do not have major safety concerns [8, 9, 11].

At what minimum BMI physicians working in primary care would consider prescribing GLP-1 RAs to patients with and without weight related comorbidities aligned with the drugs’ indications. However, respondents were more likely to consider prescribing GLP-1 RAs at a higher BMI than the indication, rather than a lower BMI. This could be due to the lack of availability of GLP-1 RAs during the time this study was conducted, making the physicians prescribe GLP-1 RAs more scarcely to secure availability for those in greatest need. It could also be explained by the high costs of treatment, where physicians and patients find the cost of GLP-1 RAs more worthwhile if the patient has a higher BMI. There was a trend where respondents considered that GLP-1 RAs should be subsidized to patients at a minimum BMI that was higher than the approved indications. However, the response distribution was mixed, likely due to there currently being no GLP-1 RAs included in the pharmaceutical benefit system for weight loss indications.

The greatest barriers for prescribing GLP-1 RAs for obesity at the time of the survey were low drug availability (86%) due to supply shortages and high costs for the patient (69%). Most respondents (87%) also considered that prescriptions of GLP-1 RAs to patients with obesity without T2DM should be limited when availability of these drugs is low. Concerns regarding drug availability reflected the supply situation at the time of survey administration in 2024. These shortages have since been resolved, although the patient cost remains high. Nevertheless, physicians in primary care are optimistic about the effectiveness and future role of GLP-1 RAs as weight loss drugs, as 91% of the respondents believe that GLP-1 RAs will play an important role in treating obesity in the future.

This study has strengths and limitations. One strength is that the survey questions were developed based on tested and reliable methods and inspired from questionnaire items from other validated surveys. Measures were taken to reduce bias of the survey, like including questionnaire items with both negative and positive formulation. The inclusion of five different Swedish regions was intended to improve the representativeness of the study population, as it includes both some of the bigger and smaller regions in Sweden. This study also has limitations, of which the main ones are a low response rate, a small sample size and uncertain denominator, non-response bias, possible regional non-representativeness, social desirability bias, lack of a validated questionnaire, limited depth of qualitative data and risks of self-selection bias. Self-selection bias refers to when respondents actively select themselves into the sample due to bias, and therefore the sample does not represent the general study population [36]. Due to the low response rate obtained in this study (28%) and the small sample size, there is a risk that the results obtained are not completely representative for the entire population studied. However, low response rates are a common challenge when conducting web-based surveys, and measures were taken to improve the quality and transparency of the reported findings from this survey study, like utilizing the CHERRIES checklist [30, 37]. Furthermore, the response rate may have been underestimated, as the denominator for the Örebro region was based on an outdated mailing list of 300 physicians. Another source indicated that only 110 physicians were working in primary care in the region at the time of the study, which would correspond to a response rate as high as 52%, rather than the estimated 19% in the region. Assuming that the second source provides a more accurate estimate, the total response rate of the study may be higher than presented.

## Conclusion

The results from this study indicate that among responding physicians in five Swedish regions, attitudes toward GLP-1Ras were generally positive but interpretation is limited by the low response rate and possible self-selection bias. At the time of the survey, limited drug availability and high patient costs were the most commonly reported barriers to prescribing GLP-1 RAs. Supply constraints have changed since the survey period, but high patient cost remains an important issue. However, the physicians in primary care think that GLP-1 RAs will play an important role in the treatment of obesity in the future. The physicians’ considerations on when to prescribe these drugs aligned with current recommendations and indications. More studies are needed in this area to further understand the attitudes towards GLP-1 RAs from a clinical perspective. Future studies should use nationally representative sampling, validated survey instruments, and linkage to prescribing data where feasible.

## Supplementary Information

Supplementary Material 1: Appendix 1. Survey translated to English using Microsoft Co-pilot (AI). Appendix 2. Detailed Description of The Recruitment Process. Appendix 3. Proportion of respondents who would prescribe various types of drugs to patients with and without comorbidities. Appendix 4. Results from analysis on differences between gender and field of work.

## Data Availability

The individual survey responses are not allowed to share publicly due to confidentiality reasons; however, upon reasonable request, additional analyses can be conducted after contact with the corresponding author.

## Abbreviations

BMI: Body mass index
DTC: Drug and therapeutics committee
ERA: Ethical Review Authority
GLP-1 RA: Glucagon-like peptide 1 receptor agonist
SMPA: Swedish Medical Products Agency
T2DM: Type 2 diabetes mellitus
WHO: World Health Organization
