# Supplementary material for: Attitudes of Swedish primary care physicians toward GLP-1 receptor agonists for overweight and obesity: a cross-sectional survey
Source: BMC Prim Care. 2026 Aug 5;27:289. doi: 10.1186/s12875-026-03488-y (PMC13445834; doi:10.1186/s12875-026-03488-y)
Supplement: Supplementary file 1 [file 12875_2026_3488_MOESM1_ESM.docx]

# Appendix

**Appendix 1. Survey translated to English using Microsoft Co-pilot (AI)

English translation of questionnaire**

**1. What is your occupation?**

*You must provide a response*

- Resident physician
- Specialist in general medicine
- Specialist in another field
- Other

**2. What is your gender identity?**

- Man
- Woman
- Other / Prefer not to say

**3. How many years have you worked as a physician?**

- 0–5 years
- 6–10 years
- 11–20 years
- More than 20 years
- Prefer not to say

**4. In which region do you primarily work at the moment?**

- Gävleborg
- Uppsala
- Västmanland
- Västernorrland
- Örebro
- None of the above

**5. In what type of healthcare setting do you primarily work?**

- Regional primary care center/health center
- Private primary care center/health center
- Other healthcare setting
- Prefer not to say

**6. How often do you encounter patients without diabetes whom you consider in need of treatment for overweight/obesity (lifestyle intervention/medication/surgery)?**

- Never
- Once a month or less
- Several times a month
- Once a week
- Several times a week
- Every day
- I don’t know

**The following questions concern obesity (BMI ≥ 30 kg/m²).**

**7. Is obesity a chronic disease?**

- Yes
- No

**8. Primary care physicians have the prerequisites to help patients with obesity achieve a healthier weight.**

- Strongly disagree
- Partly disagree
- Neither agree nor disagree
- Partly agree
- Strongly agree
- I don’t know / I have no opinion

**9. Who is responsible for ensuring that patients with obesity achieve a healthier weight?**

- The patient holds full responsibility
- The patient holds most of the responsibility
- The patient and the healthcare system share equal responsibility
- The healthcare system holds most of the responsibility
- The healthcare system holds full responsibility
- I don’t know / I have no opinion

**10. GLP-1 RAs are effective drugs for weight loss among patients who have not achieved sufficient weight loss from lifestyle changes alone.**

- Strongly disagree
- Partly disagree
- Neither agree nor disagree
- Partly agree
- Strongly agree
- I don’t know / I have no opinion

## **11. At what minimum BMI would you consider prescribing medications within the GLP-1 receptor agonist class to patients WITHOUT weight-related comorbidities?**

- BMI below 27
- BMI 27 or higher
- BMI 30 or higher
- BMI 35 or higher
- BMI 40 or higher
- I would not consider prescribing regardless of BMI
- I don’t know

## **12. At what minimum BMI would you consider prescribing medications within the GLP-1 receptor agonist class to patients WITH weight-related comorbidities?**

- BMI below 27
- BMI 27 or higher
- BMI 30 or higher
- BMI 35 or higher
- BMI 40 or higher
- I would not consider prescribing regardless of BMI
- I don’t know

## **13. At what minimum BMI do you think patients WITHOUT weight-related comorbidities should be eligible for subsidized medications within the GLP-1 receptor agonist class?**

- BMI below 27
- BMI 27 or higher
- BMI 30 or higher
- BMI 35 or higher
- BMI 40 or higher
- Medications should not be subsidized regardless of BMI
- I don’t know

## **14. At what minimum BMI do you think patients WITH weight-related comorbidities should be eligible for subsidized medications within the GLP-1 receptor agonist class?**

- BMI below 27
- BMI 27 or higher
- BMI 30 or higher
- BMI 35 or higher
- BMI 40 or higher
- Medications should not be subsidized regardless of BMI
- I don’t know

## **15. Treatment with medications within the GLP-1 receptor agonist class must continue for life to maintain a healthier weight.**

- Strongly disagree
- Partly disagree
- Neither agree nor disagree
- Partly agree
- Strongly agree
- I don’t know / I have no opinion

## **16. Assuming that all medications are available, which of the following medications would you consider prescribing to a patient with obesity WITHOUT type 2 diabetes mellitus? (Multiple answers can be selected.)**

- Xenical® (orlistat)
- Mysimba® (naltrexone/bupropion)
- Saxenda® (liraglutide)
- Victoza® (liraglutide)
- Ozempic® (semaglutide)
- Rybelsus® (semaglutide)
- Wegovy® (semaglutide)
- Mounjaro® (tirzepatide)
- Trulicity® (dulaglutide)
- Other/None of the above
- I don’t know

## **17. Assuming that all medications are available, which of the following medications would you consider prescribing to a patient with obesity AND type 2 diabetes mellitus? (Multiple answers can be selected.)**

- Xenical® (orlistat)
- Mysimba® (naltrexone/bupropion)
- Saxenda® (liraglutide)
- Victoza® (liraglutide)
- Ozempic® (semaglutide)
- Rybelsus® (semaglutide)
- Wegovy® (semaglutide)
- Mounjaro® (tirzepatide)
- Trulicity® (dulaglutide)
- Other/None of the above
- I don’t know

## **18. When the availability of medications within the GLP-1 receptor agonist class is low, prescribing these medications to patients with obesity WITHOUT type 2 diabetes mellitus should be restricted.**

- Strongly disagree
- Partly disagree
- Neither agree nor disagree
- Partly agree
- Strongly agree
- I don’t know / I have no opinion

## **19. Which of the following factors constitute barriers for you to consider prescribing medications within the GLP-1 receptor agonist class for the treatment of obesity? (Multiple answers can be selected.)**

- High cost for the patient
- High cost for the region
- Low availability of the medications
- Medications are not included in the drug committee’s recommendations
- Uncertainty about the short-term effects of the medications on weight loss
- Uncertainty about the long-term effects of the medications on weight loss
- Uncertainty about the safety of the medications
- Other/None of the above
- I don’t know

## **20. Medications within the GLP-1 receptor agonist class will likely constitute an important part of obesity treatment in the future.**

- Strongly disagree
- Partly disagree
- Neither agree nor disagree
- Partly agree
- Strongly agree
- I don’t know / I have no opinion

**Appendix 2. Detailed Description of The Recruitment Process**

In Västmanland, the heads of primary care clinics were contacted by the author via email on October 8^th^ after approval from the DTC. The invitation email was sent to a total of 28 heads of primary care clinics. The email addresses were provided by the DTC and handled with confidentiality by the author. In Gävleborg, the author contacted Hälsovalet via email on October 8^th^, who then forwarded the email to 41 heads of primary care clinics in the region on the same day. In Örebro, the chairman of the DTC forwarded the author’s invitation email directly to primary care physicians in the region on October 10^th^. The email list used consisted of around 300 people. However, another source implied that there were only 110 primary care physicians in Örebro at the time, indicating that the email invitation also reached physicians who were temporarily stationed in Örebro. In Uppsala, the author got in contact with the DTC via the help of an associate professor at Uppsala university and a clinical pharmacist co-opted in the DTC. The heads of 48 primary care clinics in Uppsala were then contacted via the DTC on October 14^th^, who forwarded the invitation email written by the author. In Västernorrland, contact physicians of primary care clinics were contacted via the author’s contact person in the DTC on October 18^th^. The invitation letter was sent to 28 contact physicians and a total of 32 different primary care units. Three of the units had no contact physician, so the email invitation was instead sent to the clinic’s function mailbox.

**Appendix 3. Proportion of respondents who would prescribe various types of drugs to patients with and without comorbidities.**

To patients with obesity with no T2DM, 77% and 72% of respondents would consider prescribing Wegovy® and Saxenda®, respectively. The corresponding proportion for Mysimba® and Xenical® are 47% and 45%, respectively. To patients with obesity and T2DM, a strong majority of respondents would consider prescribing Ozempic® (semaglutide), provided that all drugs are available, and more than half of respondents would consider prescribing Trulicity® (dulaglutide), Rybelsus® (semaglutide) and Victoza® (liraglutide) (Figure 6).

**Appendix 4. Results from analysis on differences between gender and field of work.**

***Table A1:*** *Calculated 95% confidence interval based on difference in proportions between men and women for various variables.*

| **Variable (Questionnaire Item)** | **Males with outcome (%)  (N = 61)** | **Females with outcome (%)  (N = 100)** | **Difference women - men (%)** | **95% C.I (%)** |
| --- | --- | --- | --- | --- |
| Obesity chronic disease – yes (7) | 72,1 | 86,0 | 13,9 | 0,7 - 27,0* |
| Primary care physicians can help patients with obesity – agree^a^ (8) | 67,2 | 68,0 | 0,8 | -18,8 – 20,4 |
| Primary care physicians can help patients with obesity – disagree^b^ (8) | 23,0 | 31,0 | 8,0 | -5,9 – 22,0 |
| Losing weight is the patient’s responsibility - agree^a^ (9) | 65,6 | 68,0 | 2,4 | -12,6 – 17,5 |
| Effective drugs – agree^a^ (10) | 96,7 | 96,0 | -0,7 | -6,6 – 5,2 |
| Prescribe at BMI 30 or more without comorbidities (11) | 41,0 | 54,0 | 13,0 | -2,7 – 28,8 |
| Prescribe at BMI 27 or more with comorbidities (12) | 27,9 | 45,0 | 17,1 | 2,2 – 32,0* |
| Prescribe at BMI 30 or more with comorbidities (12) | 36,1 | 34,0 | -2,1 | -17,3 – 13,1 |
| Subsidization at BMI 30 or more without comorbidities (13) | 21,3 | 28,0 | 6,7 | -6,8 – 20,2 |
| Subsidization at BMI 35 or more without comorbidities (13) | 29,5 | 27,0 | -2,5 | -16,9 – 11,9 |
| No subsidization regardless of BMI without comorbidities (13) | 19,7 | 10,0 | -9,7 | -21,3 – 1,9 |
| Subsidization at BMI 27 or more with comorbidities (14) | 13,1 | 25,0 | 11,9 | -0,1 – 23,9 |
| Subsidization at BMI 30 or more with comorbidities (14) | 29,5 | 31,0 | 1,5 | -13,1 – 16,1 |
| Subsidization at BMI 35 or more with comorbidities (14) | 14,8 | 19,0 | 4,2 | -7,5 – 16,0 |
| GLP-1 RAs require lifelong treatment – agree^a^ (15) | 54,1 | 49,0 | -5,1 | -21,0 – 10,8 |
| Limit prescribing in times of shortage – agree^a^ (18) | 88,5 | 87,0 | -1,5 | -11,9 – 8,8 |
| GLP-1 RAs important in the future – agree^a^ (20) | 90,2 | 91,0 | 0,8 | -8,5 – 10,2 |

* = *P < 0.05* and considered statistically significant

*a =* proportion who answered either agree or strongly agree (‘top 2 boxes’)

b = proportion who answered either disagree or strongly disagree (‘bottom 2 boxes’)

***Table A2:*** *Calculated 95% confidence interval based on difference in protportions between primary care physicians working in regional and private primary care for* *for various variables.*

| **Variable (Questionnaire Item)** | **Regional with outcome (%)  (N = 98)** | **Private with outcome (%)  (N = 65)** | **Difference regional – private (%)** | **95% C.I (%)** |
| --- | --- | --- | --- | --- |
| Obesity chronic disease – yes (7) | 77,6 | 86,2 | -8,6 | -20,4 – 3,2 |
| Primary care physicians can help patients with obesity – agree^a^ (8) | 70,4 | 64,6 | 5,8 | -8,9 – 20,5 |
| Primary care physicians can help patients with obesity – disagree^b^ (8) | 24,5 | 16,9 | 7,6 | -4,9 – 20,0 |
| Losing weight is the patient’s responsibility - agree^a^ (9) | 61,2 | 75,4 | -14,2 | -28,4 – 0,1 |
| Effective drugs – agree^a^ (10) | 98,0 | 93,8 | 4,1 | -2,4 – 10,6 |
| Prescribe at BMI 30 or more without comorbidities (11) | 52,0 | 46,2 | 5,9 | -9,8 – 21,5 |
| Prescribe at BMI 27 or more with comorbidities (12) | 39,8 | 36,9 | 2,9 | -12,3 – 18,1 |
| Prescribe at BMI 30 or more with comorbidities (12) | 34,7 | 35,4 | -0,7 | -15,7 – 14,3 |
| Subsidization at BMI 30 or more without comorbidities (13) | 27,6 | 24,6 | 2,9 | -10,8 – 16,6 |
| Subsidization at BMI 35 or more without comorbidities (13) | 27,6 | 27,7 | -0,1 | -14,2 – 13,9 |
| No subsidization regardless of BMI without comorbidities (13) | 12,2 | 15,4 | -3,1 | -14,1 – 7,8 |
| Subsidization at BMI 27 or more with comorbidities (14) | 24,5 | 15,4 | 9,1 | -3,1 – 21,3 |
| Subsidization at BMI 30 or more with comorbidities (14) | 31,6 | 29,2 | 2,4 | -12,0 – 16,8 |
| Subsidization at BMI 35 or more with comorbidities (14) | 15,3 | 20,0 | - 4,7 | -16,8 – 7,4 |
| GLP-1 RAs require lifelong treatment – agree^a^ (15) | 30,6 | 29,2 | 1,4 | -13,0 – 15,7 |
| Limit prescribing in times of shortage – agree^a^ (18) | 85,7 | 87,7 | -2,0 | -12,6 – 8,6 |
| GLP-1 RAs important in the future – agree^a^ (20) | 90,8 | 90,8 | 0,0 | -11,9 – 12,0 |

* = *P < 0.05* and considered statistically significant

*a =* proportion who answered either agree or strongly agree (‘top 2 boxes’)

b = proportion who answered either disagree or strongly disagree (‘bottom 2 boxes’)
